# Investigating Unique Environmental Contributions to the Neural Representation of Written Words: A Monozygotic Twin Study

**DOI:** 10.1371/journal.pone.0031512

**Published:** 2012-02-08

**Authors:** Joonkoo Park, Denise C. Park, Thad A. Polk

**Affiliations:** 1 Department of Psychology, University of Michigan, Ann Arbor, Michigan, United States of America; 2 Center for Vital Longevity, University of Texas at Dallas, Dallas, Texas, United States of America; Hôpital Robert Debré, France

## Abstract

The visual word form area (VWFA) is a region of left inferior occipitotemporal cortex that is critically involved in visual word recognition. Previous studies have investigated whether and how experience shapes the functional characteristics of VWFA by comparing neural response magnitude in response to words and nonwords. Conflicting results have been obtained, however, perhaps because response magnitude can be influenced by other factors such as attention. In this study, we measured neural activity in monozygotic twins, using functional magnetic resonance imaging. This allowed us to quantify differences in unique environmental contributions to neural activation evoked by words, pseudowords, consonant strings, and false fonts in the VWFA and striate cortex. The results demonstrate significantly greater effects of unique environment in the word and pseudoword conditions compared to the consonant string and false font conditions both in VWFA and in left striate cortex. These findings provide direct evidence for environmental contributions to the neural architecture for reading, and suggest that learning phonology and/or orthographic patterns plays the biggest role in shaping that architecture.

## Introduction

The left lateral occipitotemporal cortex has been identified as a critical site for the visual processing of written words [1], [2]. Brain imaging experiments collectively demonstrate that the middle portion of the left occipitotemporal sulcus bordering the fusiform gyrus and the inferior temporal gyrus exhibits greater neural activation in response to written words compared to other control stimuli in a variety of tasks [for review see 3,4]. Although there is debate over whether this region is specialized for word forms, it is often referred to as the visual word form area (VWFA) [1].

The location of the VWFA is quite consistent across individuals and cultures [1], [5], [6], which suggests that some innate mechanisms play a role in the development of this neural architecture. On the other hand, reading is a recent development on an evolutionary time scale, it is not shared with other species, and it does not develop without extensive experience. It is therefore unlikely that our brain has been genetically programmed, via natural selection, to process written words specifically [3], [7], [8].

Whether and how experience shapes the VWFA has been tested in a variety of different ways in previous studies. In some studies, VWFA activation in response to a known script versus an unknown script was compared [9], [10], [11], [12], [13]. If experience with visual word forms influences the neural signature of VWFA, then one would expect differential neural activation levels between known and unknown scripts. The results, however, have been mixed. For example, Baker et al. [9] reported greater VWFA activation in response to a known script than an unknown script (own language vs. foreign language) while Xue et al. [13] reported the opposite.

Other studies examined patterns of VWFA activation as a function of word regularity or frequency [12], [14], [15]. The idea behind these studies is that VWFA activation will be modulated by the frequency with which we encounter word forms. However, there is no clear consensus from these studies either. On the one hand, orthographic regularity (stimuli composed of more frequent letter bigrams and trigrams) has been found to increase VWFA activity [12], [14]. On the other hand, VWFA activity has been reported to decrease as a function of word frequency [15].

The reasons for such inconsistent findings are largely unknown. One thing we do know, however, is that factors such as attentional engagement, task difficulty, and time on task play critical roles in the magnitude of neural activation [16], [17], [18], [19]. Therefore, subtle differences in tasks and other experimental parameters can easily influence response magnitude and may therefore obscure the results making it difficult to examine experience-dependent effects in VWFA. So, how else can we empirically test the role of experience in shaping the neural architecture for written words?

Twin studies make it possible to directly assess the amount of genetic and environmental contributions in explaining individual differences in a trait. In particular, monozygotic (MZ) twins make it possible to quantify the effect of unique environmental factors on a trait. Because MZ twins reared together share all their genetic alleles and potential common environmental effects, the correlation of a phenotypic trait in MZ twins provides an estimate of variability explained by these common factors (genetics and common environment) [20]. Extending this reasoning, a trait that is more influenced by unique environmental factors will result in smaller MZ correlations relative to a trait that is less susceptible to such influences.

In the present study, we examined neural activity in the VWFA in MZ twins in order to study how the unique environment that we experience over time shapes this brain region. We measured VWFA activity evoked by words and wordlike stimuli using functional magnetic resonance imaging (fMRI). Assuming that the neural representation of written words is influenced by experience, we expected to find greater environmental effects (smaller MZ correlations) on the neural response to words compared to unfamiliar wordlike stimuli. Furthermore, we tested whether such environmental effects are also evident in other visual areas or are specific to the VWFA.

Of course, reading is a complicated process that involves multiple subcomponents including visual, orthographic, phonological, and semantic processing. We therefore included stimuli that make differential demands on these subprocesses (false fonts, consonant strings, pseudowords, words) in order to investigate at what stage(s) unique environmental effects have their effects. False fonts require visual processing, but do not involve letters. Consonant strings involve letters, but are not pronounceable or orthographically regular. Pseudowords are pronounceable and orthographically regular, but are not semantically meaningful. And real words involve letters, are pronounceable and orthographically regular, and are semantically meaningful. By examining MZ twins' neural response to all four stimulus types, we hoped to identify the stage of processing at which unique environmental factors have their greatest effect.

## Methods

### Participants

Sixteen MZ pairs (7 male pairs, ages 18–29 with median age of 22.5) participated in the study. All participants were screened to ensure they were right-handed, native English speakers, psychologically and physically healthy, not taking medications with psychotropic or vascular effects, and free of any other MRI safety contraindications. Zygosity was determined by comparing up to fifteen genetic markers (D3S1358, TH01, D21S11, D18S51, Penta E, D5S818, D13S317, D7S820, D16S539, CSF1PO, Penta D, vWA, D8S1179, TPOX, FGA) from the buccal cells of twins collected by swabbing the cheek of each participant. Twins in whom all the markers matched were classified as monozygotic. All study procedures were reviewed and approved by the Institutional Review Boards at the University of Texas at Dallas, the University of Texas Southwestern Medical School, and the University of Michigan. All participants provided detailed written consent prior to their involvement in the study.

### Stimulus Materials

**Figure 1:**
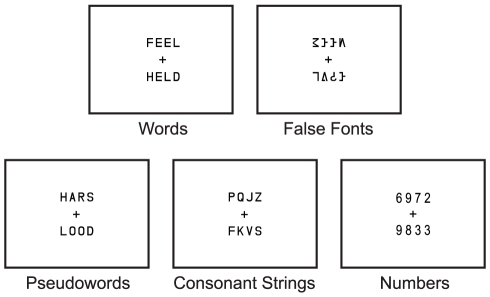
Examples of stimuli used in this study. Monozygotic twin participants performed a visual matching task on pairs of real words (WD), pseudowords (PW), consonant strings (CS), numbers (NB), and false fonts (FF) and judged whether the two items were the same or different.

Words (WD) were randomly chosen from the MCWord database (Medler & Binder, 2005, MCWord: An On-Line Orthographic Database of the English Language, http://www.neuro.mcw.edu/mcword) with word frequency ranging from 205.4 to 497.3 per million. Pseudowords, or pronounceable nonwords, were created from constrained trigram-based strings from the MCWord database. Consonant strings were random combinations of consonants. False fonts (FF) were adapted from Vinckier et al. [12]. These false fonts were designed to be visually similar to upper case letters. Additionally, random combinations of Arabic numbers (NB) were included, which served as a contrast when functionally identifying the VWFA. All strings were composed of four characters (mono-spaced typeface with 2° visual angle in height), and only capital letters were used (see Fig. 1).

### Procedure

The fMRI experiment consisted of five 5-minute runs with eighteen 16-sec blocks, pseudorandomly ordered. Each run consisted of three blocks of each of the five stimulus categories in addition to three blocks of fixation viewing. Each block consisted of 8 trials (1.5 sec of presentation and 0.5 sec of inter-trial interval). On each trial, two strings from the same stimulus category were presented 4.2° above and below the central cross as shown in Figure 1. Participants judged whether the two strings were the same or different. The correct answer was “same” in half of all the trials. All visual stimuli were presented via E-prime (Psychology Software Tools, Pittsburgh, PA) and displayed by a back-projection system. Participants made responses using buttons under the right index and middle fingers (Lumina response pad; Cedrus, San Pedro, CA).

### Data Acquisition

Brain images were acquired with a Philips Achieva 3T whole-body scanner at UT Southwestern using the Philips SENSE parallel acquisition technique. Functional scans were acquired as axial slices, with a voxel size of 3.4 mm×3.4 mm×3.5 mm. At each of 148 BOLD acquisitions per run, 43 axial slices were acquired (covering the whole brain; TR = 2.0 s, TE = 25 ms). A high-resolution axial T1 MPRAGE was acquired (voxel size 1 mm isotropic; TR = 8.27 ms, TE = 3.82 ms).

### Activation Analysis and Inter-individual Registration

The first step in the analysis involved estimating the neural response within each participant to each experimental condition. Functional data were processed using SPM5 (Wellcome Department of Cognitive Neurology, London, UK, http://www.fil.ion.ucl.ac.uk). The functional images underwent slice-timing correction and realignment to the mean volume. Then, activations in response to each stimulus (i.e. WD, PW, CS, FF and NB) relative to fixation were estimated using the standard general linear model (GLM) with a high-pass filter with a cut-off frequency at 1/128 Hz and correcting for temporal autocorrelation with an AR(1) model. The model included separate regressors for each of the experimental conditions in each run convolved with a canonical hemodynamic response function, as well as six nuisance covariates modeling head translation and rotation. In order to use independent data to define the region of interest and to test the effect of interest, the neural activations were estimated separately for odd and even runs. This procedure resulted in volumetric brain maps of parameter estimates (beta values from the GLM) from odd and even runs for each of the five categories in each participant.

In order to minimize the contribution of brain morphology in estimating the similarity of activation maps in twin pairs, a cortex-based inter-individual registration technique was used by incorporating the FreeSurfer 4.5 (Martinos Center for Biomedical Imaging, http://surfer.nmr.mgh.harvard.edu) automated reconstruction stream. First, each participant's T1 anatomical image was coregistered with the mean functional image. Then, this image underwent a series of reconstruction streams in FreeSurfer, which resulted in the identification of gray/white matter boundaries and gyral/sulcal folding patterns. Inter-individual registration was performed using this surface-based atlas by mapping individual cortical folding patterns to the FreeSurfer average curvature map. This procedure allows direct alignment of the anatomy instead of image intensities. The resulting surface map consisted of 163,842 vertices on each hemisphere.

In the next step, the functional brain maps from each individual were mapped onto the average surface map. First, individual volumetric parameter estimate maps computed from the functional data analysis were mapped onto individual surface maps. Then, these individual surface maps were mapped onto the FreeSurfer average surface map. The resulting maps were surface-smoothed using a Gaussian kernel with 6 mm full-width-half-maximum.

### Regions of Interest

**Figure 2:**
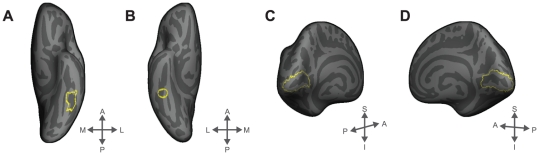
The regions of interest (in yellow) on an inflated surface: the visual word form area (VWFA) (A), the right homologue of VWFA or the right OTS (B), the left striate cortex (C), and the right striate cortex (D). A: anterior; P: posterior; M: medial; L: lateral; S: superior; and I: inferior.

The VWFA was functionally defined from the second-level random-effects group analysis on the surface maps of WD+PW+CS>NB from even runs (*p*<10^−5^, uncorrected; extent >50 mm^2^) (Table 1). This contrast resulted in one contiguous region in the left fusiform and inferior temporal area subtending 831 vertices (approximately 470 mm^2^) (see Fig. 2A).

**Table 1:** Cluster results of the second-level random-effects group analysis of WD+PW+CS>NB (*p*<10^−5^, uncorrected) for defining the VWFA.

Coordinates (Talairach)	Maximum t-value	Size (mm^2^)
−39.6 −42.9 −14.3	6.402	470.88
−42.0 3.0 20.7	5.648	27.59
−44.1 −32.9 −16.8	5.391	31.39
−39.7 −20.6 −17.6	5.344	10.58
−51.6 −40.4 7.2	5.241	7.49

No suprathreshold activation was observed in the right hemisphere.

We also examined the bilateral striate cortices. The striate ROIs were defined anatomically from the FreeSurfer cortical parcellation scheme (lh.V1.label and rh.V1.label) (see Fig. 2C and 2D).

In addition, we selected the right homologue of the VWFA (right occipito-temporal sulcus or OTS) as a control region, within the visual cortex, where we expected little environmental influence as a function of experimental conditions. This region was defined anatomically by constructing an 8-mm sphere around the approximate coordinate opposite from the VWFA peak [40.62, −41.46, −13.29] on the FreeSurfer average surface space (see Fig. 2B).

### Monozygotic Twin Approach

The goal of the MZ twin analysis was to quantify the amount of total phenotypic variance in VWFA activity explained by unique environmental effects. MZ twins reared together share all of their genetic alleles (A) and common environment (C), so any differences between MZ twins can be attributed to unique environment effects (E). That is, P = A+C+E, and the intraclass correlation (ICC) between MZ twins becomes the proportion of phenotypic variation accounted for by genetics and shared environment (Var(A+C)/Var(P)). Therefore, the complement of this MZ correlation (1−Var(E)/Var(P)) represents the proportion of phenotypic variance explained by unique environmental effects. Note that the unique environmental effect also includes variance accounted for by measurement error, and it is assumed in this study that this unsystematic error variance is comparable across the four conditions.

### Parameter Estimates and MZ Correlations

**Figure 3:**
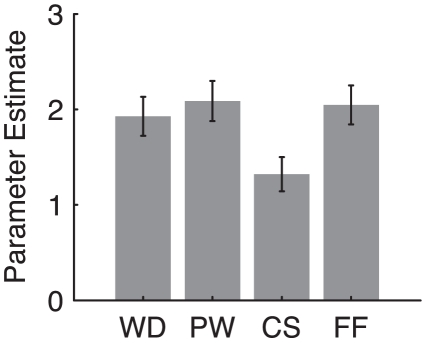
The mean response magnitude in the VWFA. Error bars represent standard error across all participants.

**Figure 4:**
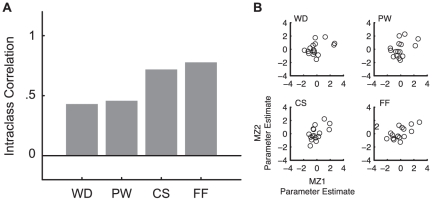
*Z*-transformed ICC estimates in the VWFA (A), and scatter plots of the parameter estimates between MZ twins (B). ICC_FF_ was greater than ICC_WD_ (*p* = 0.029), there was a significant linearity in the ICC across the four conditions (*p* = 0.010), and ICC_CS_ and ICC_FF_ together was greater than ICC_WD_ and ICC_PW_ together (*p* = 0.008).

Parameter estimates for the WD, PW, CS, and FF conditions from odd runs within each ROI were computed for each participant. Then, the mean parameter estimate in each condition was computed across all participants (see Fig. 3 and Fig. S1) and used as the primary dependent measure. In order to quantify the effect of unique environment, the ICC between MZ twins was computed (see Fig. 4). First, linear effects of age and sex were removed from the parameter estimates to remove any variance explained by these covariates (i.e., the residuals from a regression model including age and sex as regressors were treated as the measure of interest in the MZ correlation analysis). Then, the ICC across MZ twins of the mean parameter estimate for each condition was computed [21]. To enable comparison between different correlation estimates, the computed ICC underwent Fisher's *r*-to-*z* transformation. The MZ correlation (or MZ ICC) reported in this paper refers to *z*-transformed ICC. ICC_WD_, ICC_PW_, ICC_CS_, and ICC_FF_ refer to ICC estimates in each of the four conditions.

### Statistical Significance

We were primarily interested in testing two a priori hypotheses of interest: first that the ICC_FF_ is significantly greater than ICC_WD_ and second, that there is a significant increase in ICC across the four conditions. The statistical significance of these effects of interest (and other post-hoc tests) was tested using a permutation method. The null hypothesis is that there is no difference between ICC_CS_, ICC_FF_, ICC_WD_, and ICC_PW_ (i.e., stimulus type has no effect on ICC). If the null hypothesis is true, then permuting the condition labels would not affect the results. We therefore computed a null distribution of statistical values (ICC_FF_−ICC_WD_ for the difference between ICC_FF_ and ICC_WD_, and (3/4)×ICC_FF_+(1/4)×ICC_CS_−(1/4)×ICC_PW_−(3/4)×ICC_WD_ for the linear contrast) based on different permutations of condition labels. For example, say in one twin pair, parameter estimates for the WD condition were 1 and 2 and parameter estimates for the FF condition were 5 and 6. In each permutation, the labeling of the conditions was rearranged randomly. So, in one case, parameter estimates 1 and 2 would be labeled as the FF condition and 5 and 6 would be labeled as the WD condition. In another case, parameter estimates 1 and 2 would be labeled as the WD condition and 5 and 6 would be labeled as the FF condition. In each of the 10,000 repetitions, this kind of random permutation was done in each twin pair separately. Then, the null distribution was constructed from 10,000 of these *permuted* estimates. Finally, the significance of the *observed* estimate (based on the correct labeling of the conditions) was compared against the null distribution of 10,000 values. If the observed value was larger than 95% of the values in the null distribution, then it was considered significant.

### ICC Map

The VWFA, as well as our other ROIs, may contain subregions that perform different functions, so we also investigated whether ICC varies within an ROI. To do so, we applied a statistical method that is able to capture patterns of ICC measures across a spatial dataset [22]. This spatial decomposition method provides an effective ICC estimate at each cortical point (just like computing a voxelwise ICC estimate) while using the information from spatial dependencies within an ROI to achieve better control of noise typical of fMRI data [see 22 for more details].

We visualized patterns of ICC estimates within the VWFA and the striate cortex using this spatial decomposition method. The basis images of each ROI were constructed by computing eigenimages of all the WD+PW+CS>FF contrast images from the 32 subjects. These basis images provide information about the spatial dependencies within each ROI. Then, the spatial decomposition method was applied to create an ICC map. The ICC map allowed us to visually inspect whether subregions within an ROI have different ICC estimates.

## Results

### Behavioral Results

Reaction times and accuracy in response to each experimental condition were analyzed (Table 2). There was a significant difference between the reaction times across conditions tested by a within-subject ANOVA design (*F*_2.72, 84.42_ = 96.320, *p*<0.001, Greenhouse-Geisser corrected). A post-hoc contrast analysis showed that this difference was mainly driven by slower RT for NB than CS (*F*_1,31_ = 9.164, *p* = 0.005) and slower RT for FF than NB (*F*_1,31_ = 204.456, *p*<0.001). The same test for accuracy showed a significant difference between the conditions (*F*_3.00, 93.05_ = 14.074, *p*<0.0001, Greenhouse-Geisser corrected), and this difference was mainly driven by lower accuracy in the false fonts condition than the other conditions (A post-hoc contrast of FF<NB showed *F*_1,31_ = 27.983, *p*<0.001).

**Table 2:** Behavioral results of the visual matching task for each experimental condition performed in the scanner.

	Word	Pseudoword	Consonant Strings	Number Strings	False Fonts
Accuracy (%)	98.9(1.25)	98.2(2.02)	98.2(2.16)	98.7(1.98)	96.6(2.64)
Reaction Time (ms)	659.05(84.57)	663.66(85.31)	670.78(103.61)	678.28.60(89.18)	781.23(111.88)

Mean accuracy and median reaction time for the correct trials were measured for each MZ twin (*N* = 32), and the average (standard deviations in parentheses) of these scores across subjects are reported.

### Response Magnitude

Many previous studies have compared neural response magnitude evoked by familiar words to unfamiliar words (e.g., foreign words or false fonts) in VWFA (Fig. 2A). In order to directly compare our results to these previous studies, mean activation levels in the VWFA were computed as shown in Figure 3. As we expected, there was a significant difference in the response magnitude across the four experimental conditions in the VWFA (*F*_2.03, 62.93_ = 18.335, *p*<0.001, Greenhouse-Geisser corrected within-subject ANOVA). This effect was driven by relatively smaller response magnitude in the CS condition compared to the other conditions (*F*_1,31_ = 84.641, *p*<0.001). There was no difference across the WD, PW, and FF conditions (*F*_1.42, 43.99_ = 0.837, *p* = 0.404, Greenhouse-Geisser corrected within-subject ANOVA).

A smaller response magnitude in the CS condition compared to the WD and PW conditions is consistent with previous studies showing hierarchical organization of VWFA [12] and sensitivity to bigram frequency [14]. However, the finding of a comparable response in the FF condition compared to the WD and PW conditions is different from a previous study that used the same set of false font stimuli [12]. Moreover, we found no region in the bilateral ventral visual cortex showing greater activation in the WD condition relative to the FF condition (*p*<10^−5^, uncorrected; extent >50 mm^2^). These differences in response magnitude may have been driven by differences in the tasks. See Text S1 and Figure S1 in the Supporting Information for the response magnitude in all the ROIs and their reliability measures.

### Unique Environmental Effects in VWFA

ICC estimates between MZ twins' VWFA activation are shown in Figure 4A. As predicted, there was a monotonic increase in ICC across the four conditions, with the smallest in ICC_WD_ and the greatest in ICC_FF_. The difference between ICC_FF_ and ICC_WD_ was statistically significant (effect size = 0.246, *p* = 0.029). This pattern is consistent with our primary hypothesis of greater unique environmental effects in the neural activity associated with familiar real word processing compared to unfamiliar false font processing in VWFA.

A linear contrast across the four conditions was also statistically significant (effect size = 0.232, *p* = 0.010), consistent with the idea that unique environmental effects become larger as the stimuli become more word-like. We noticed, however, that ICC_WD_ and ICC_PW_ were similar to each other while ICC_CS_ and ICC_FF_ were similar to each other. Indeed, a post-hoc contrast analysis showed a significant difference between ICC_CS_ and ICC_FF_ together and ICC_WD_ and ICC_PW_ together (i.e. a contrast of (1/2)×ICC_FF_+(1/2)×ICC_CS_−(1/2)×ICC_PW_−(1/2)×ICC_WD_; effect size = 0.217, *p* = 0.008).

The scatter plots of the parameter estimates between MZ twins shown in Figure 4B show a much tighter correlation between MZ activation in the FF condition than in the WD condition as well as a monotonic trend toward tighter correlation across the conditions. Note also that these effects in ICC estimates cannot be explained by the effects of response magnitude (see Fig. 3).

One might ask whether differences in ICC measures could arise from differences in processing strategies. For example, the WD task could potentially be performed using different strategies (e.g. relying on semantics, phonology, or visual form) while the FF task would seem to require reliance on visual form and might therefore be less susceptible to individual differences in strategy. Could this difference explain our finding that ICC is greater in the FF condition that the WD condition? If so, the same effect should occur even if we analyze unrelated pairs of people rather than MZ twins. However, when unrelated subjects were randomly paired up (across 10,000 repetitions) and their ICC was estimated, there was no sign of differences in ICC estimates across the four conditions. Mean, median, and std of the z-transformed ICC's were as follows: WD, mean = −0.0308, median = −0.0411, std = 0.270; PW, mean = −0.0374, median = −0.0486, std = 0.270; CS, mean = −0.0361, median = −0.0416, std = 0.272; FF, mean = −0.0367, median = −0.0379, std = 0.275. Also, if differences in ICC measures arise from differences in processing strategies, then one might expect systematic differences in ICC estimates in reaction times across the conditions. The ICC estimates in reaction time of correct trials were 0.505 (WD), 0.560 (PW), 0.413 (CS), and 0.558 (FF). A permutation test of statistical significance showed that none of the pair-wise differences were significant (*p*>0.450, two-tailed). Thus, the observed differences in ICC across conditions in the twins cannot be attributed to strategy differences between the conditions.

### ICC in the Striate Cortex and the Right OTS

**Figure 5:**
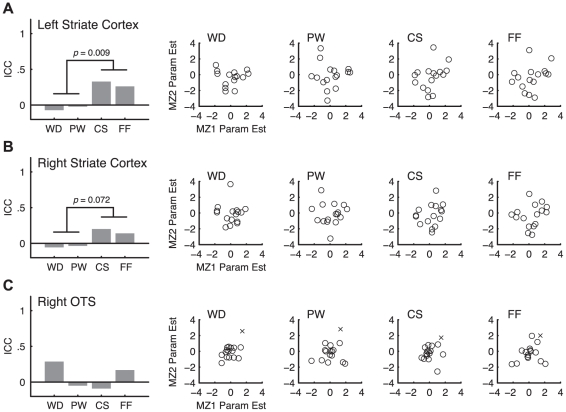
*Z*-transformed ICC estimates and scatter plots in the left striate cortex (A), right striate cortex (B), and the right OTS (C).

We also examined whether similar ICC patterns are found in the left and right striate cortex. In the left striate cortex (Fig. 5A), the patterns of ICC were similar to those in the VWFA. Specifically, there was a trend showing greater ICC_FF_ than ICC_WD_ (*p* = 0.057), and ICC_CS_ and ICC_FF_ together were significantly greater than ICC_WD_ and ICC_PW_ (*p* = 0.009). In the right striate cortex (Fig. 5B), the effects were weaker, but there was again a trend toward ICC_FF_ being greater than ICC_WD_ (*p* = 0.171) and ICC_CS_ and ICC_FF_ together being greater than ICC_WD_ and ICC_PW_ (*p* = 0.072).

In addition, we tested whether such patterns in ICC exist in the right homologue of VWFA, namely the right OTS. In this region, there was no clear pattern in the ICC estimates (Fig. 5C). There was no significant difference between ICC_WD_ and ICC_FF_ (*p* = 0.717, two-tailed), and none of the rest of the pair-wise differences were significant (*p*>0.286, two-tailed). Note that the bar graph in Figure 5C and the statistics in text is based on ICC estimates excluding one influential outlier as shown as an “x” in the scatter plot in Figure 5C. However, including this outlier does not change the results qualitatively. Even with this outlier, there is no difference between ICC_FF_ and ICC_WD_ in the right OTS (*p* = 0.302, two-tailed) and none of the pair-wise comparisons reach significance (*p*>0.286, two-tailed).

### ICC Maps

**Figure 6:**
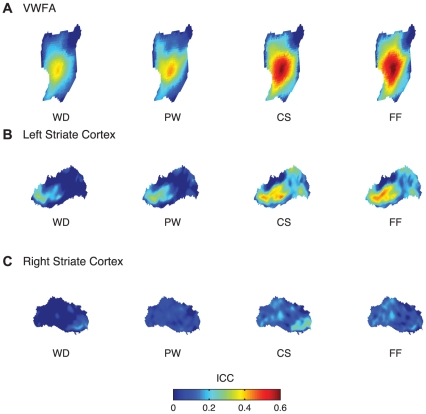
ICC maps in the VWFA (A), the left striate cortex (B), and the right OTS (C). See Figure 2 for the precise location of these ROI's in the context of the whole brain.

Spatial maps of ICC estimates in the VWFA are shown in Figure 6A. Consistent with the ICC measured from the mean response magnitude values (Fig. 4A), ICC_FF_ was greater than ICC_WD_ and there was a linear trend across the four conditions. Differences in the ICC values were most apparent in the middle portion of the VWFA, and there were no conspicuous subregions within the VWFA that carried different ICC estimates.

In the left striate cortex, ICC estimates were greater in the posterior region of the ROI in general (Fig. 6B). In addition, visual inspection of the ICC maps across the four conditions showed that this posterior region showed the greatest differences in ICC values, suggesting that the environmental effect is most pronounced in this occipital pole area. In the right striate cortex, there were few obvious differences across the four conditions (Fig. 6C), although the CS and FF conditions seemed to result in greater ICC measures across the ROI in general.

## Discussion

In this study, we investigated how the environment influences the functional organization of the visual word form area (VWFA). Neural activations in response to words, pseudowords, consonant strings, and false fonts were measured in monozygotic twins, and the proportion of phenotypic variability explained by unique environmental effects was estimated. The results showed greater unique environmental contributions to neural activity evoked by words compared to false fonts, confirming the idea that the development of VWFA must be partially experientially-driven. More importantly, there was a greater effect of unique environment on words and pseudowords than on consonant strings and false fonts. These findings suggest that the neural responsivity of the VWFA is not entirely “hard-wired” and is affected by the impact of experience. Moreover, the impact of experience in the VWFA increases as stimuli become more word-like.

There are two influential theoretical claims regarding the role of experience in shaping the neural architecture for written words. One theory proposes that neurons in the left occipitotemporal region, which are predisposed to processing fine-grain visual features, become tuned to encode abstract representations of visual word forms [7], [23]. Another theory argues that neurons in this occipitotemporal region become responsive to words (and possibly to other stimuli as well) due to unique top-down feedback connections from phonological and semantic processing areas [24], [25]. While the underlying mechanism for the development of the so-called VWFA is explained differently in the two theories, there are some common predictions that they make regarding the neural response in VWFA to different types of orthographic stimuli.

One prediction from both theories is that the neural response in VWFA will be greater when viewing a known script than an unknown script. This simple prediction has been tested in many previous studies using various stimuli and tasks. The results to date, however, have been somewhat mixed. One study demonstrated greater left occipitotemporal activation for native words compared to foreign words [9], while another study demonstrated greater activation for foreign words compared to native words [13]. Another study found a non-significant difference between the two [26], and yet another reported that false fonts activated more than random letter sequences but less than real words in this region [12]. Lastly, one study showed greater activation for foreign words than native words in the more lateral region of the occipitotemporal cortex but the opposite pattern in the more medial region [11].

Inconsistent results are also apparent in studies that examine VWFA activity as a function of the frequency with which we encounter orthographic stimuli. Binder et al. [14] found that the putative VWFA region is sensitive to bigram frequency, showing greater activation in response to letter strings with more frequent bigrams. Vinckier et al. [12] also found that this region is hierarchically organized showing greater activation in response to more orthographically regular letter strings. On the other hand, Kronbichler et al. [15] found decreasing putative VWFA activation as a function of increasing word frequency even when bigram frequency was controlled for.

In this study, we addressed these issues by studying monozygotic twins (MZ), which makes it possible to quantify the role of environment in explaining differences between individuals. MZ twins are genetically identical so any differences in their behaviors must be due to different experiences and can be estimated by computing MZ correlation. The fact that we observed greater MZ correlation in the false font condition compared to the word condition suggests that environment plays a significantly stronger role in shaping the VWFA activation in response to words than false fonts.

We also found a monotonically increasing influence of unique environmental effects as stimuli became more word-like, with the correlations decreasing from false fonts and consonant strings to pseudowords and words in the VWFA. This finding suggests that there are greater unique environmental contributions as more subcomponents of reading are involved. This linear contrast was mainly driven by the difference between words and pseudowords on the one hand, and consonant strings and false fonts on the other.

What distinguishes words and pseudowords from consonant strings and false fonts? Perhaps the most obvious difference is that words and pseudowords are pronounceable whereas consonant strings and false fonts are not. Our results are therefore consistent with the hypothesis that experience with phonological processing plays a significant role in shaping the response of the VWFA.

Training studies have found some evidence consistent with this phonological hypothesis. Xue et al. [13] studied how visual, phonological, and semantic training in an artificial language influenced VWFA activity and reported that VWFA activity increased after phonological training. Similarly, Hashimoto & Sakai [11] trained subjects to match an artificial symbol with a sound that could either be a speech sound or a nonspeech sound, and Brem et al. [27] trained children to map letters and speech sounds. In both studies, the authors' found that VWFA activity selectively increased as a result of training, suggesting that associated phonological processing drives the development of VWFA. Consistent with these previous studies, we found that the effects of unique experience are larger for stimuli that are pronounceable and that therefore involve phonological processing. Furthermore, our results show an effect of long-term experience with a real language, not short-term training in an artificial language.

Another characteristic that distinguishes words and pseudowords from consonant strings and false fonts is orthographic regularity. That is, words and pseudowords are composed of frequent letter sequences and their spelling conforms to the orthographic rules of English. In contrast, consonant strings involve infrequent letter sequences that violate the spelling conventions of English (e.g., failing to follow *q* with *u*, or including *j* in a consonant cluster) and are thus orthographically irregular. And of course, false fonts do not involve letters at all and are therefore also not orthographically regular. The finding that the effects of unique environment are greater for words and pseudowords is therefore also consistent with the hypothesis that experience processing orthographically regular letter strings plays a significant role in shaping the response of the VWFA [12], [14]. And of course, the phonological hypothesis and the orthographic regularity hypothesis are not mutually exclusive; both could be true.

The same patterns that we observed in the VWFA were also found in left striate cortex but not in the right occipito-temporal sulcus. More interestingly, patterns of ICC estimates in the striate cortex revealed that the environmental modulation of neural activity is most pronounced around the left occipital pole. These findings imply that years of experience with words modulate neural activity not only in VWFA but also in early visual areas, as also suggested in a recent study showing the modulation of the left V1 activity by literacy [10].

Because reading is an acquired skill, it is unlikely that there is a genetic predisposition to preferentially process one of these stimulus types over another (even if there are genetic influences on some of the underlying perceptual mechanisms). Imagine a person who has absolutely no knowledge of English letters (or any other similar Roman letters). It would presumably be impossible for that person to tell whether an item presented in our experiment was a word, pseudoword, consonant string, or a string of false fonts. Consequently, the differences in MZ correlation observed in our study must be due to differences in environmental effects on the four conditions. Furthermore, the observed differences among MZ correlations cannot be explained by overall response magnitude effects (Fig. 3). This study, therefore, provides a unique way to investigate environmental effects on neural activity, overcoming limitations in previous studies where interpretations are based exclusively on response magnitude.

Unique environmental factors are known to explain substantial phenotypic variance in personality, psychopathology, and cognition [28]. In fact, although it may seem surprising, shared environmental influences that are common to all members of a family seem to be far less important than unique, nonshared environmental influences [29]. Put simply, common experiences like being raised by the same parents and attending the same school have less influence than experiences that are unique to an individual [30]. We speculate that the major nonshared influence on the neural architecture of reading is an individual's personal experience with reading (e.g., what they read, how often they read), above and beyond how they were taught in school. There are two reasons. First, MZ twins typically attend the same classes in elementary school and would therefore receive very similar reading instruction, meaning that most reading instruction would be a common, shared experience, not an experience that is unique to each individual. Second, recall that the correlations were smaller (implying larger nonshared environmental effects) for words and pseudowords than for consonant strings and false fonts. Words and pseudowords are pronounceable and orthographically regular whereas consonant strings and false fonts are not. We therefore hypothesize that it is experience seeing and pronouncing orthographically regular stimuli that produces the observed effects. And of course, most of that experience comes from one's personal experience with reading. Furthermore, we note that exactly what someone reads, and when, is largely (though certainly not entirely) specific to that individual, rather than being common to both twins.

At the same time, it should also be noted that ICC estimates in VWFA were greater than zero (WD, *p* = 0.060; PW, *p* = 0.049; CS, *p* = 0.006; FF, *p* = 0.003; two-tailed; these statistics were computed from an assumption that a function of ICC (r), [ = r/sqrt((1−r^2^)/(N−2))], is distributed approximately as *t* with *df* = N−2), suggesting that a significant portion of the phenotypic variance can be explained by common factors, possibly genetics. Considering that dyslexia is significantly heritable [31] and that reading makes demands on general perceptual processes likely to be influenced by genetics, these findings are not surprising. All we can say based on the present results is that common factors (either genetics or shared environment) are playing a significant role in shaping the response of the VWFA, but that they become less important, and unique experience becomes more important, as the stimuli become more word-like.

In sum, the present findings provide direct evidence about how experience shapes the neural processing of written words. They overcome limitations of previous studies that interpret data based exclusively on response magnitude and suggest that learning phonology and/or orthographic patterns (or both) makes the largest contribution in shaping the neural response of the VWFA.

## Supporting Information

Figure S1 The mean response magnitude in the right homologue of VWFA or the right OTS (A), the left striate cortex (B), and the right striate cortex (C). In the right OTS (A), the response magnitude across the four conditions differed significantly (*F*_2.70, 83.79_ = 29.017, *p*<0.001, Greenhouse-Geisser corrected within-subject ANOVA), and a post-hoc contrast revealed that this effect was mainly driven greater response magnitude in the FF condition compared to the three other conditions (*F*_1,31_ = 57.764, *p*<0.001) and greater response magnitude in the WD and PW condition compared to the CS condition (*F*_1,31_ = 9.394, *p* = 0.004). In the left striate cortex (B), the response magnitude across the four conditions differed significantly (*F*_2.62, 81.07_ = 8.632, *p*<0.001, Greenhouse-Geisser corrected within-subject ANOVA), and a post-hoc contrast revealed that this effect was mainly driven greater response magnitude in the FF condition compared to the three other conditions (*F*_1,31_ = 20.993, *p*<0.001). In the right striate cortex (C), the response magnitude across the four conditions differed significantly (*F*_2.79, 86.41_ = 6.931, *p*<0.001, Greenhouse-Geisser corrected within-subject ANOVA), and a post-hoc contrast revealed that this effect was mainly driven greater response magnitude in the FF condition compared to the three other conditions (*F*_1,31_ = 16.845, *p*<0.001) and greater response magnitude in the WD and PW condition compared to the CS condition (*F*_1,31_ = 4.151, *p* = 0.050).
(DOCX)

Text S1 In order to make inference from different ICC values across the conditions, it is critical to confirm that any differences in ICC are not driven by differences in reliability measures. We therefore computed the split-half reliability (even and odd runs) of the four conditions in four ROIs. In the VWFA, the reliability estimates were 0.679 (WD), 0.857 (PW), 0.661 (CS), and 0.733 (FF) in four conditions. Using a permutation test as described below, we tested for any significant pair-wise differences in these reliability estimates. Two-tailed p-values for pair-wise differences were *p* = 0.629 (FF vs CS), *p* = 0.852 (FF vs PW), *p* = 0.652 (FF vs WD), *p* = 0.507 (CS vs PW), *p* = 0.974 (CS vs WD), and *p* = 0.533 (PW vs WD). In the right OTS, the reliability estimates were 0.736 (WD), 0.510 (PW), 0.345 (CS), and 0.828 (FF), and none of the pair-wise differences were statistically significant (*p*>0.177). Seemingly low reliability measures in the right OTS, for example in the PW and CS conditions, were due to an outlying subject. Excluding this one subject resulted in reliability measures of 0.810 (PW) and 0.777 (CS). In the left striate cortex, the reliability estimates were 0.858 (WD), 0.799 (PW), 0.797 (CS), and 0.901 (FF), and none of the pair-wise differences were statistically significant (*p*>0.577). In the right striate cortex, the reliability estimates were 0.893 (WD), 0.711 (PW), 0.743 (CS), and 0.863 (FF), and none of the pair-wise differences were statistically significant (*p*>0.863).
(DOCX)
