# Supplementary material for: Investigating Unique Environmental Contributions to the Neural Representation of Written Words: A Monozygotic Twin Study
Source: PLoS One. 2012 Feb 8;7(2):e31512. doi: 10.1371/journal.pone.0031512 (PMC3275550; doi:10.1371/journal.pone.0031512)
Supplement: Figure S1 — The mean response magnitude in the right homologue of VWFA or the right OTS (A), the left striate cortex (B), and the right striate cortex (C). In the right OTS (A), the response magnitude across the four conditions differed significantly (F 2.70, 83.79 = 29.017, p<0.001, Greenhouse-Geisser corrected within-subject ANOVA), and a post-hoc contrast revealed that this effect was mainly driven greater response magnitude in the FF condition compared to the three other conditions (F 1,31 = 57.764, p<0.001) and greater response magnitude in the WD and PW condition compared to the CS condition (F 1,31 = 9.394, p = 0.004). In the left striate cortex (B), the response magnitude across the four conditions differed significantly (F 2.62, 81.07 = 8.632, p<0.001, Greenhouse-Geisser corrected within-subject ANOVA), and a post-hoc contrast revealed that this effect was mainly driven greater response magnitude in the FF condition compared to the three other conditions (F 1,31 = 20.993, p<0.001). In the right striate cortex (C), the response magnitude across the four conditions differed significantly (F 2.79, 86.41 = 6.931, p<0.001, Greenhouse-Geisser corrected within-subject ANOVA), and a post-hoc contrast revealed that this effect was mainly driven greater response magnitude in the FF condition compared to the three other conditions (F 1,31 = 16.845, p<0.001) and greater response magnitude in the WD and PW condition compared to the CS condition (F 1,31 = 4.151, p = 0.050). (DOCX) [file pone.0031512.s001.docx]

**Supporting Information**

|  |  |  |
| --- | --- | --- |

Figure S1. The mean response magnitude in the right homologue of VWFA or the right OTS (A), the left striate cortex (B), and the right striate cortex (C).

In the right OTS (A), the response magnitude across the four conditions differed significantly (*F*_2.70, 83.79_ = 29.017, *p* < 0.001, Greenhouse-Geisser corrected within-subject ANOVA), and a post-hoc contrast revealed that this effect was mainly driven greater response magnitude in the FF condition compared to the three other conditions (*F*_1,31_ = 57.764, *p* < 0.001) and greater response magnitude in the WD and PW condition compared to the CS condition (*F*_1,31_ = 9.394, *p* = 0.004).

In the left striate cortex (B), the response magnitude across the four conditions differed significantly (*F*_2.62, 81.07_ = 8.632, *p* < 0.001, Greenhouse-Geisser corrected within-subject ANOVA), and a post-hoc contrast revealed that this effect was mainly driven greater response magnitude in the FF condition compared to the three other conditions (*F*_1,31_ = 20.993, *p* < 0.001).

In the right striate cortex (C), the response magnitude across the four conditions differed significantly (*F*_2.79, 86.41_ = 6.931, *p* < 0.001, Greenhouse-Geisser corrected within-subject ANOVA), and a post-hoc contrast revealed that this effect was mainly driven greater response magnitude in the FF condition compared to the three other conditions (*F*_1,31_ = 16.845, *p* < 0.001) and greater response magnitude in the WD and PW condition compared to the CS condition (*F*_1,31_ = 4.151, *p* = 0.050).
