# Supplementary material for: Investigating Unique Environmental Contributions to the Neural Representation of Written Words: A Monozygotic Twin Study
Source: PLoS One. 2012 Feb 8;7(2):e31512. doi: 10.1371/journal.pone.0031512 (PMC3275550; doi:10.1371/journal.pone.0031512)
Supplement: Text S1 — In order to make inference from different ICC values across the conditions, it is critical to confirm that any differences in ICC are not driven by differences in reliability measures. We therefore computed the split-half reliability (even and odd runs) of the four conditions in four ROIs. In the VWFA, the reliability estimates were 0.679 (WD), 0.857 (PW), 0.661 (CS), and 0.733 (FF) in four conditions. Using a permutation test as described below, we tested for any significant pair-wise differences in these reliability estimates. Two-tailed p-values for pair-wise differences were p = 0.629 (FF vs CS), p = 0.852 (FF vs PW), p = 0.652 (FF vs WD), p = 0.507 (CS vs PW), p = 0.974 (CS vs WD), and p = 0.533 (PW vs WD). In the right OTS, the reliability estimates were 0.736 (WD), 0.510 (PW), 0.345 (CS), and 0.828 (FF), and none of the pair-wise differences were statistically significant (p>0.177). Seemingly low reliability measures in the right OTS, for example in the PW and CS conditions, were due to an outlying subject. Excluding this one subject resulted in reliability measures of 0.810 (PW) and 0.777 (CS). In the left striate cortex, the reliability estimates were 0.858 (WD), 0.799 (PW), 0.797 (CS), and 0.901 (FF), and none of the pair-wise differences were statistically significant (p>0.577). In the right striate cortex, the reliability estimates were 0.893 (WD), 0.711 (PW), 0.743 (CS), and 0.863 (FF), and none of the pair-wise differences were statistically significant (p>0.863). (DOCX) [file pone.0031512.s002.docx]

**Supporting Information**

Text S1. In order to make inference from different ICC values across the conditions, it is critical to confirm that any differences in ICC are not driven by differences in reliability measures. We therefore computed the split-half reliability (even and odd runs) of the four conditions in four ROIs. In the VWFA, the reliability estimates were 0.679 (WD), 0.857 (PW), 0.661 (CS), and 0.733 (FF) in four conditions. Using a permutation test as described below, we tested for any significant pair-wise differences in these reliability estimates. Two-tailed p-values for pair-wise differences were *p* = 0.629 (FF vs CS), *p* = 0.852 (FF vs PW), *p* = 0.652 (FF vs WD), *p* = 0.507 (CS vs PW), *p* = 0.974 (CS vs WD), and *p* = 0.533 (PW vs WD). In the right OTS, the reliability estimates were 0.736 (WD), 0.510 (PW), 0.345 (CS), and 0.828 (FF), and none of the pair-wise differences were statistically significant (*p* > 0.177). Seemingly low reliability measures in the right OTS, for example in the PW and CS conditions, were due to an outlying subject. Excluding this one subject resulted in reliability measures of 0.810 (PW) and 0.777 (CS). In the left striate cortex, the reliability estimates were 0.858 (WD), 0.799 (PW), 0.797 (CS), and 0.901 (FF), and none of the pair-wise differences were statistically significant (*p* > 0.577). In the right striate cortex, the reliability estimates were 0.893 (WD), 0.711 (PW), 0.743 (CS), and 0.863 (FF), and none of the pair-wise differences were statistically significant (*p* > 0.863).
